# Supplementary material for: Cyclosporin A as an Add-On Therapy to a Corticosteroid-Based Background Treatment in Patients with COVID-19: A Multicenter, Randomized Clinical Trial
Source: J Clin Med. 2024 Sep 4;13(17):5242. doi: 10.3390/jcm13175242 (PMC11396137; doi:10.3390/jcm13175242)
Supplement: Supplementary file 1 [file jcm-13-05242-s001.zip › Suppl file 3 MOD.pptx]

## Slide 1
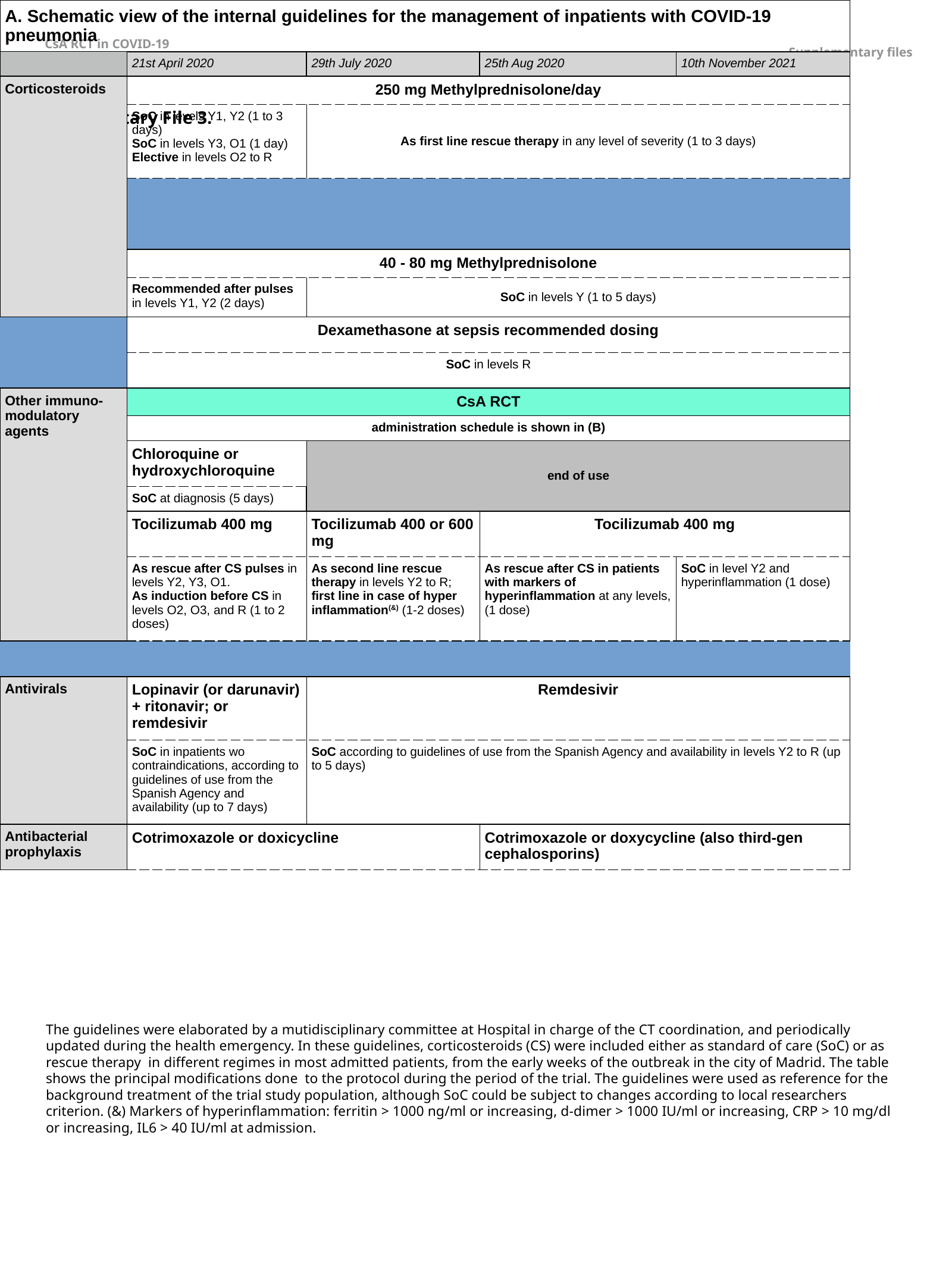

CsA RCT in COVID-19
Supplementary files
Supplementary File 3.
| A. Schematic view of the internal guidelines for the management of inpatients with COVID-19 pneumonia | | | | |
| --- | --- | --- | --- | --- |
| | 21st April 2020 | 29th July 2020 | 25th Aug 2020 | 10th November 2021 |
| Corticosteroids | 250 mg Methylprednisolone/day | | | |
| | SoC in levels Y1, Y2 (1 to 3 days) SoC in levels Y3, O1 (1 day) Elective in levels O2 to R | As first line rescue therapy in any level of severity (1 to 3 days) | | |
| | | | | |
| | | | | |
| | 40 - 80 mg Methylprednisolone | | | |
| | Recommended after pulses in levels Y1, Y2 (2 days) | SoC in levels Y (1 to 5 days) | | |
| | Dexamethasone at sepsis recommended dosing | | | |
| | SoC in levels R | | | |
| Other immuno- modulatory agents | CsA RCT | | | |
| | administration schedule is shown in (B) | | | |
| | Chloroquine or hydroxychloroquine | end of use | | |
| | SoC at diagnosis (5 days) | | | |
| | Tocilizumab 400 mg | Tocilizumab 400 or 600 mg | Tocilizumab 400 mg | |
| | As rescue after CS pulses in levels Y2, Y3, O1. As induction before CS in levels O2, O3, and R (1 to 2 doses) | As second line rescue therapy in levels Y2 to R; first line in case of hyper inflammation(&) (1-2 doses) | As rescue after CS in patients with markers of hyperinflammation at any levels, (1 dose) | SoC in level Y2 and hyperinflammation (1 dose) |
| | | | | |
| Antivirals | Lopinavir (or darunavir) + ritonavir; or remdesivir | Remdesivir | | |
| | SoC in inpatients wo contraindications, according to guidelines of use from the Spanish Agency and availability (up to 7 days) | SoC according to guidelines of use from the Spanish Agency and availability in levels Y2 to R (up to 5 days) | | |
| Antibacterial prophylaxis | Cotrimoxazole or doxicycline | | Cotrimoxazole or doxycycline (also third-gen cephalosporins) | |
| | SoC in inpatients wo contraindications (5 days) | | SoC in inpatients wo contraindications (5 - 7 days) | |
| Thrombo-prophylaxis | LMWH | | | |
| | SoC in all inpatient groups (different schedules according to risk levels) | | | |
The guidelines were elaborated by a mutidisciplinary committee at Hospital in charge of the CT coordination, and periodically updated during the health emergency. In these guidelines, corticosteroids (CS) were included either as standard of care (SoC) or as rescue therapy in different regimes in most admitted patients, from the early weeks of the outbreak in the city of Madrid. The table shows the principal modifications done to the protocol during the period of the trial. The guidelines were used as reference for the background treatment of the trial study population, although SoC could be subject to changes according to local researchers criterion. (&) Markers of hyperinflammation: ferritin > 1000 ng/ml or increasing, d-dimer > 1000 IU/ml or increasing, CRP > 10 mg/dl or increasing, IL6 > 40 IU/ml at admission.

## Slide 2
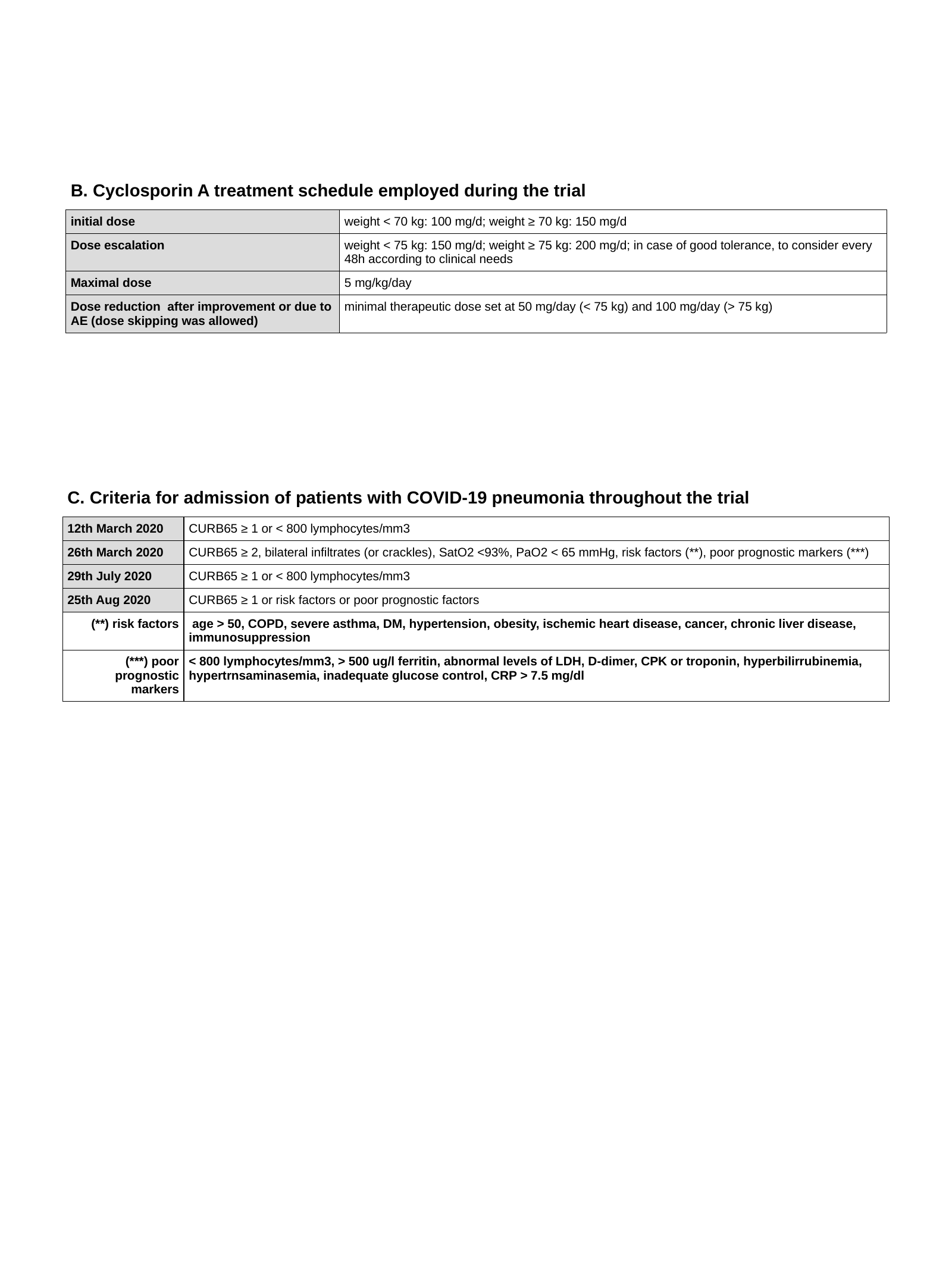

| B. Cyclosporin A treatment schedule employed during the trial | |
| --- | --- |
| initial dose | weight < 70 kg: 100 mg/d; weight ≥ 70 kg: 150 mg/d |
| Dose escalation | weight < 75 kg: 150 mg/d; weight ≥ 75 kg: 200 mg/d; in case of good tolerance, to consider every 48h according to clinical needs |
| Maximal dose | 5 mg/kg/day |
| Dose reduction after improvement or due to AE (dose skipping was allowed) | minimal therapeutic dose set at 50 mg/day (< 75 kg) and 100 mg/day (> 75 kg) |
| C. Criteria for admission of patients with COVID-19 pneumonia throughout the trial | |
| --- | --- |
| 12th March 2020 | CURB65 ≥ 1 or < 800 lymphocytes/mm3 |
| 26th March 2020 | CURB65 ≥ 2, bilateral infiltrates (or crackles), SatO2 <93%, PaO2 < 65 mmHg, risk factors (\*\*), poor prognostic markers (\*\*\*) |
| 29th July 2020 | CURB65 ≥ 1 or < 800 lymphocytes/mm3 |
| 25th Aug 2020 | CURB65 ≥ 1 or risk factors or poor prognostic factors |
| (\*\*) risk factors | age > 50, COPD, severe asthma, DM, hypertension, obesity, ischemic heart disease, cancer, chronic liver disease, immunosuppression |
| (\*\*\*) poor prognostic markers | < 800 lymphocytes/mm3, > 500 ug/l ferritin, abnormal levels of LDH, D-dimer, CPK or troponin, hyperbilirrubinemia, hypertrnsaminasemia, inadequate glucose control, CRP > 7.5 mg/dl |
