# Supplementary material for: Cyclosporin A as an Add-On Therapy to a Corticosteroid-Based Background Treatment in Patients with COVID-19: A Multicenter, Randomized Clinical Trial
Source: J Clin Med. 2024 Sep 4;13(17):5242. doi: 10.3390/jcm13175242 (PMC11396137; doi:10.3390/jcm13175242)
Supplement: Supplementary file 1 [file jcm-13-05242-s001.zip › Suppl file 4 mod.pptx]

## Slide 1
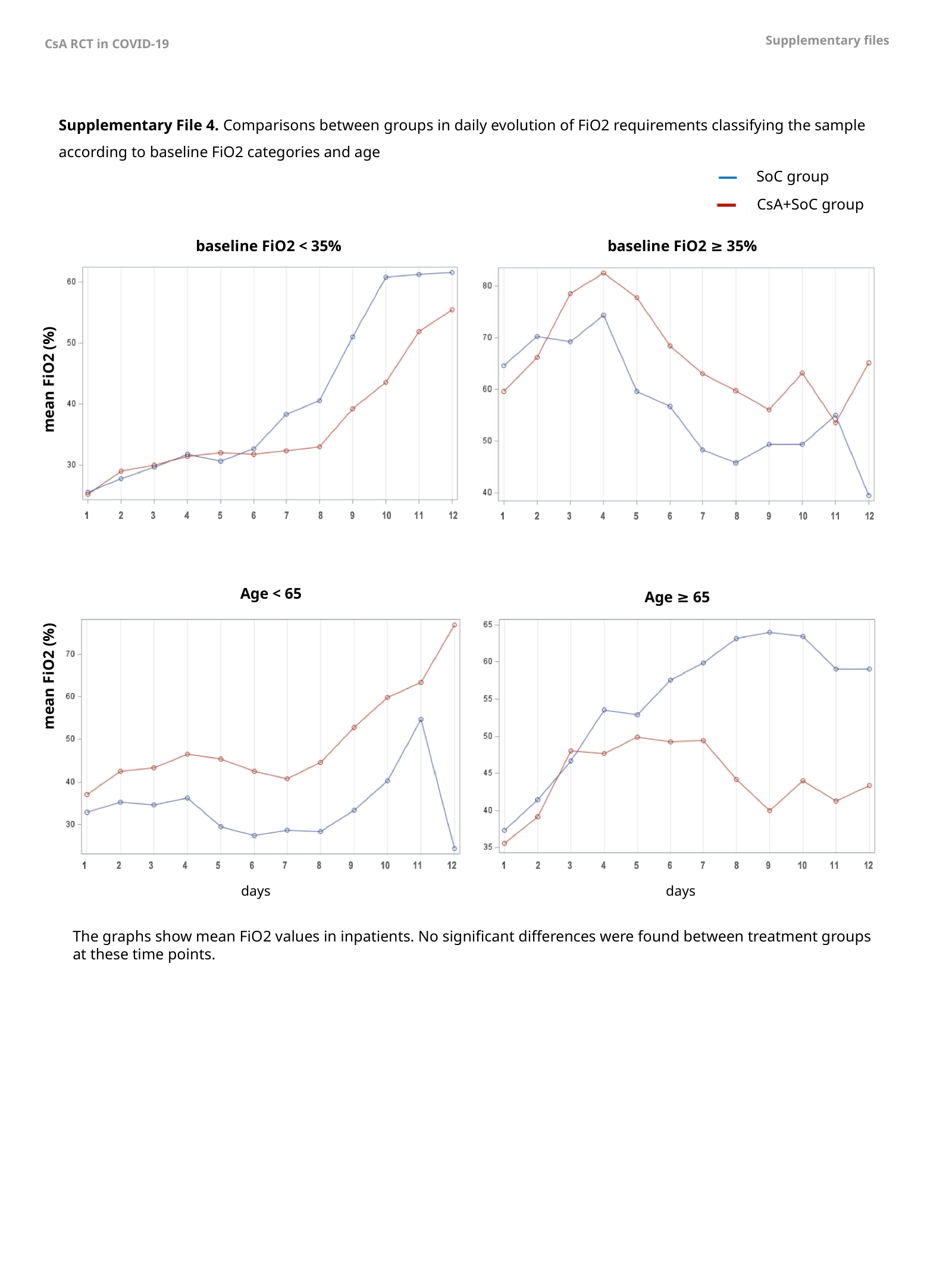

Supplementary files
CsA RCT in COVID-19
Supplementary File 4. Comparisons between groups in daily evolution of FiO2 requirements classifying the sample according to baseline FiO2 categories and age
SoC group
CsA+SoC group
baseline FiO2 < 35%
baseline FiO2 ≥ 35%
mean FiO2 (%)
Age < 65
Age ≥ 65
mean FiO2 (%)
days
days
The graphs show mean FiO2 values in inpatients. No significant differences were found between treatment groups at these time points.
