# Supplementary material for: Cyclosporin A as an Add-On Therapy to a Corticosteroid-Based Background Treatment in Patients with COVID-19: A Multicenter, Randomized Clinical Trial
Source: J Clin Med. 2024 Sep 4;13(17):5242. doi: 10.3390/jcm13175242 (PMC11396137; doi:10.3390/jcm13175242)
Supplement: Supplementary file 1 [file jcm-13-05242-s001.zip › Suppl file 5.pptx]

## Slide 1
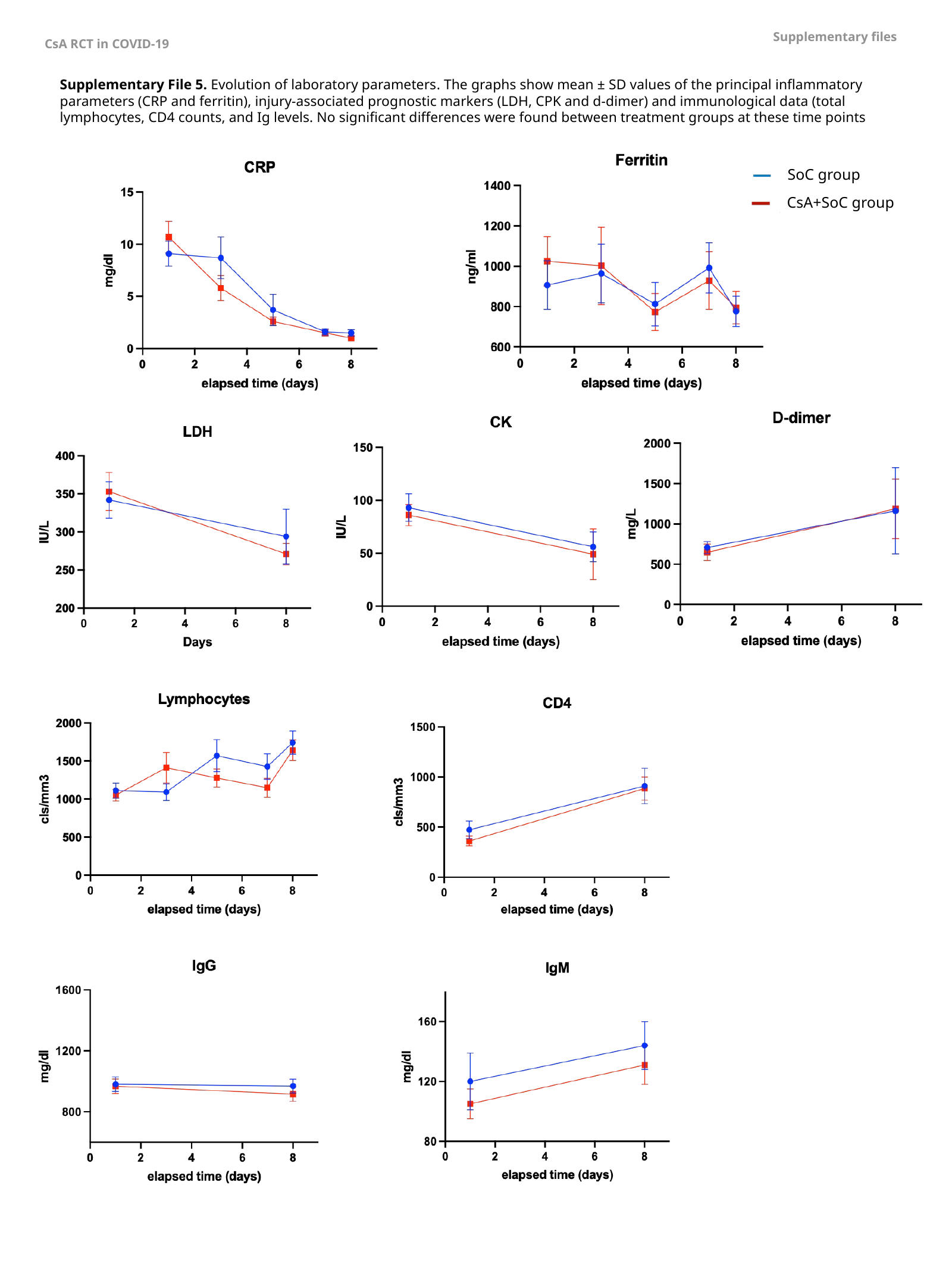

Supplementary files
CsA RCT in COVID-19
Supplementary File 5. Evolution of laboratory parameters. The graphs show mean ± SD values of the principal inflammatory parameters (CRP and ferritin), injury-associated prognostic markers (LDH, CPK and d-dimer) and immunological data (total lymphocytes, CD4 counts, and Ig levels. No significant differences were found between treatment groups at these time points
SoC group
CsA+SoC group
