# Supplementary material for: Cyclosporin A as an Add-On Therapy to a Corticosteroid-Based Background Treatment in Patients with COVID-19: A Multicenter, Randomized Clinical Trial
Source: J Clin Med. 2024 Sep 4;13(17):5242. doi: 10.3390/jcm13175242 (PMC11396137; doi:10.3390/jcm13175242)
Supplement: Supplementary file 1 [file jcm-13-05242-s001.zip › Supplementary File 1.pdf]

Supplementary file 1: Trial eligibility criteria

1. **Inclusion criteria:** Patients must meet ALL of the following criteria to be included in the study:

- Females and males aged 18 years or older.
- Clinical diagnosis of COVID19 (to be subsequently confirmed by PCR or by specific Ac isotype IgM and/or IgG) and with admission criteria according to institutional protocol.
- Acceptance and signature of the consent for the study after having received the appropriate information.

2. **Exclusion criteria:** Patients who meet ANY of the following criteria will not be eligible to participate in this study:

- Known allergy or hypersensitivity to any of the medications included in the treatment arms or to any of their components.
- Contraindication to the use of any of the medications included in the treatment arms:
  - CsA: IR EST 4.5 (GFR <30 ml/min according to the Cockroft-Gault formula).
  - Antimalarials (Chloroquine, hydroxychloroquine): Retinopathy, Myasthenia gravis.
  - Lopinavir/ritonavir: Severe liver failure.
  - Remdesivir, darunovir-ritonavir
  - Doxycycline, Azithromycin
- Renal failure (Stages 4 and 5: GFR < 30 ml/min according to the Cockroft-Gault formula).
- Decompensated liver disease (Child-Pugh stages B or C) or chronic infection with chronic B virus infection
- Pregnancy or lactation
- ~~Age> 75 years old*~~
- Participants in another clinical trial with medicinal product in the 28 days prior to the start of recruitment. Participation in observational studies is permitted.
- Refusal to participate.
- Poor health or nutritional status that in the opinion of the investigator has sufficient severity criteria to interfere with the conduct of the study or its conclusions.
- Inability of the patient, in the investigator's judgement, to understand or comply with study procedures.
- Patients aged < 50 years in the absence of sufficient severity criteria, determined by CURB65 >1 or status >A1 or presence of comorbidities.*

*Changes in eligibility criteria in protocol relevant amendment in may 2020 (~~deleted~~; added)
