# Supplementary material for: Cyclosporin A as an Add-On Therapy to a Corticosteroid-Based Background Treatment in Patients with COVID-19: A Multicenter, Randomized Clinical Trial
Source: J Clin Med. 2024 Sep 4;13(17):5242. doi: 10.3390/jcm13175242 (PMC11396137; doi:10.3390/jcm13175242)
Supplement: Supplementary file 1 [file jcm-13-05242-s001.zip › Supplementary File 2.pdf]

Supplementary File 2. FiO2 risk-based severity classification

The table shows classification of respiratory failure according to oxygen requirement and levels of hospitalization. This classification was adopted from the British Thoracic Society Guideline and employed to assess the evolution of the condition during the trial.

**Yellow levels: Ward**	**Y1**	FiO2 requirements ≤ 35% supplied by nasal cannula
	**Y2**	FiO2 requirements between 40% and 60% supplied by venturi mask
	**Y3**	FiO2 requirements ≥ 60% provided by a closed system
**Orange levels: Respiratory care Unit**	**O1**	High-flow nasal cannula (HFNC) with FiO2 < 50% and flow < 30 per min
	**O2**	High-flow nasal cannula (HFNC) with FiO2 > 50% and flow > 30 per min
	**O3**	CPAP or non-invasive ventilation with Helmet
**Red levels: Intensive care Unit**	**R1**	Invasive mechanical ventilation (IMV) with PaFi ≥ 200
	**R2**	Invasive mechanical ventilation (IMV) with PaFi < 200
	**R3**	IMV and sepsis (qSOFA 2 of 3) and/or multi-organ failure
