# Supplementary material for: Cyclosporin A as an Add-On Therapy to a Corticosteroid-Based Background Treatment in Patients with COVID-19: A Multicenter, Randomized Clinical Trial
Source: J Clin Med. 2024 Sep 4;13(17):5242. doi: 10.3390/jcm13175242 (PMC11396137; doi:10.3390/jcm13175242)
Supplement: Supplementary file 1 [file jcm-13-05242-s001.zip › Supplementary File 6.pdf]

Supplementary file 6. Independent risk factors predicting long hospital stays in the study population

Variable	Risk	ROC AUC	Cut-off
Female	OR 0.3 CI95 [0.11, 0.82] p 0.018		
Days from onset	6.8 ± 1 (vs 12 ± 2.5) p 0.025	0.284 ± 0.098 CI95 [0.092, 0.476] p 0.029	
FiO2 on day 1	32 ± 2 (vs 24 ± 1) p < 0.001	0.829 ± 0.07 CI95 [0.691, 0.967] p 0.001	26% (66% sensitivity, 91% specificity)
CRP on day 0 (mg/dL)	15.1 ± 1.3 (vs 6.5 ± 1.4) p < 0.001		
CRP on day 1 (mg/dL)	10.8 ± 1.1 (vs 4.7 ± 1) p < 0.001		
LDH on day 0 (IU/L)	374 ± 23 (vs 268 ± 23) p < 0.01		
CK (IU/dL) on day 0	100 + 11 (vs 59 ± 8) p < 0.005		
Leukocyte count on day 0 (cell/mm3)	9908 ± 858 (vs 6730 ± 559) p < 0.003		
IgG (gr/dL) on day 0	905 ± 34 (vs 1110 ± 87) p < 0.038		

Univariate analysis of independent variables. Comparisons between subgroups of patients with and stays without longer than 7 days are shown (mean ± SD). The strength of the associations between days from onset and FiO2 at day 1 with long stays was assessed with receiver operating characteristics area under the curve analysis, and cut-off values for predicting a long stay was calculated for FiO2.
