# Supplementary material for: Cyclosporin A as an Add-On Therapy to a Corticosteroid-Based Background Treatment in Patients with COVID-19: A Multicenter, Randomized Clinical Trial
Source: J Clin Med. 2024 Sep 4;13(17):5242. doi: 10.3390/jcm13175242 (PMC11396137; doi:10.3390/jcm13175242)
Supplement: Supplementary file 1 [file jcm-13-05242-s001.zip › Supplementary File 7.pdf]

Supplementary file 7. Amendments to the protocol after trial start

As of May 2020, a relevant amendment to the study protocol was submitted to Spanish Medicines Agency and Ethics Committee for evaluation, that received approval in July 2020. A summary of the key changes and their justification is described below:

1. Study population: eligibility criteria were modified to allow the inclusion of patients over 75 years of age who, in the opinion of the investigator, could participate in the study. Patients > 80 years would be stratified as severe. Patients < 50 years in situation ≤A1 and CURB≤1 without comorbidities would not be eligible. Patients included up to that moment belonged to the mild spectrum, and it is considered that this does not adequately reflect the real spectrum of severity of the pandemic and the prognostic factors identified. The following information was included in section 1.7 of the introduction: *We have analyzed the age-adjusted mortality rate in 1000 patients hospitalized at the Fundación Jiménez Díaz between March and April. These data reflect a 12.5% mortality rate in patients between 61 and 70 years of age and 29% in patients between 71 and 80 years of age; while above 81 years of age, the mortality rate reached 54%. Age was the main predictor of death among our patients, followed by severity on the CURB65 scale on admission, severity status A2 or higher, male sex and comorbidities.*
2. Study treatment allocation: For stratification purposes, patients over 80 years of age would be considered as severe, regardless of their classification according to oxygen needs.
3. Study follow-up period: time until the End-of-Study visit was extended to harmonize the time at which the assessment is made, taking as a reference the date of initial admission, so that in non severe and severe patients EOS would be performed 4 and 6 weeks from admission, respectively. In the initial version of the protocol. patients in the control group were assessed 15 days after discharge, and those in the treatment group 15 days after the end of treatment, which resulted in a large difference in some cases.
4. Study Outcomes: the main outcome variable is changed from "*proportion of patients in non severe category at 12 days of treatment*" to "*proportion of patients without oxygen support (or who have returned to baseline in the case of patients with OCD prior to admission) at 12 days of treatment, without relapses during follow-up*"·. That is, to meet the OP, the patient is required to have not relapsed during the follow-up period, which is set at a minimum of 1 month from admission and up to 45 days in patients with prolonged admission. Relapses are defined by one of the following: hospital readmission for any cause, increase in FiO2, death, acute vascular event.
5. Statistical considerations: The interim analysis was firstly scheduled to be performed when 40% of patients had reached day 7 of admission, and it was moved to day 8, at which time analytical determinations were also carried out as a part of the following period.

In addition, data from the *** (omitted for manuscript blinding) COVID Register including all patients admitted in our Institution with respiratory failure during the first wave of SARS-CoV2's pandemic (unpublished) showed that oxygen requirements predicted death risk with an AUC of 0.72 [CI95: 0.67; 0.77], and FiO_2 levels > 30.5% showing 53% sensitivity and 83% specificity for fatal events.
